# Identification of an Immunoglobulin Paratope Binding to Keratan Sulfate and Expression of a Single-Chain Derivative for Imaging

**DOI:** 10.3390/biom15020178

**Published:** 2025-01-25

**Authors:** Burak Boyraz, Rudolf Tauber, Jens Dernedde

**Affiliations:** 1 Institut für Laboratoriumsmedizin, Klinische Chemie und Pathobiochemie, Charité–Universitätsmedizin Berlin, Corporate Member of Freie Universität Berlin, Humboldt-Universität zu Berlin and Berlin Institute of Health, Augustenburger Platz 1, 13353 Berlin, Germany; boyrab92@zedat.fu-berlin.de; 2 Fachbereich Biologie, Chemie, Pharmazie, Freie Universität Berlin, Arnimallee 22, 14195 Berlin, Germany

**Keywords:** ScFv, antibody, paratope, keratan sulfate, glycosaminoglycans, imaging, MZ15, recombinant expression, immunohistochemistry

## Abstract

Keratan sulfate (KS) is a negatively charged carbohydrate linked to proteins. Several KS-bearing structural glycosaminoglycans participate to maintain the homeostasis of a functional extracellular matrix. Dysfunction of its biochemical composition and structure might therefore lead to pathological situations. For this reason, imaging of KS in tissues is an important diagnostic tool. Here, we describe the identification of the KS paratope derived from the ancestral anti-KS IgG mAb MZ15, as well as the engineering, functional recombinant expression in *E. coli*, and purification of an anti-KS single-chain variable fragment (ScFv). The ScFv enabled in vitro imaging of KS in cryosections of rat cornea by immunofluorescence microscopy comparable to the ancestral IgG MZ15.

## 1. Introduction

The extracellular matrix (ECM) is a complex and dynamic macromolecular network of proteins and polysaccharides. It constitutes a noncellular scaffold within tissues and plays an important role in establishing and maintaining the structural and functional integrity of the cellular milieu [1,2]. Its primary constituents are structural proteins, including collagens, fibronectin, laminins, and elastin, along with proteoglycans (PGs) [3]. PGs are composed of negatively charged carbohydrates, known as glycosaminoglycans (GAGs), which covalently attach to a protein core through either *N*- or *O*-linkages. GAGs are classified into major groups, which include heparin, heparan sulfate (HS), keratan sulfate (KS), chondroitin sulfate (CS), dermatan sulfate (DS), and hyaluronic acid, the latter not being associated with a protein core [4,5,6,7,8,9]. GAGs have a significant diversity in monosaccharide composition, size, and modification. The quantity and arrangement of sulfate and carboxyl groups play a crucial role, giving the distinctive strong negative charge and respective protein binding specificity of GAGs [10,11].

Besides their essential role in physiological processes, GAGs and PGs also participate in the development and progression of various inherited and acquired diseases like developmental disorders, cancer, inflammation, fibrosis, and disturbed wound healing, reflected by changes in the structure, composition, and hence physical properties of GAGs [1,12,13,14]. Therefore, the identification and characterization of alterations in the biochemical composition, localization, and expression of GAGs in various pathological conditions are of high diagnostic interest.

KS is distinguished from the other GAGs by its unique composition of repeating units of Gal*β*1–4Glc*N*Ac*β*1–3 units (*N*-acetyllactosamine) [15,16]. Keratan sulfate proteoglycans (KSPGs) are widely distributed within the body and play essential roles in different biological processes, including collagen fibrillogenesis, neural development, and regenerative processes, such as wound healing [17,18,19,20,21,22,23]. KSPGs are predominantly found in the cornea of the eye [24]. Within the corneal structure, one of several functions involves the maintenance of the regular spacing of collagen fibrils to sustain corneal transparency [25]. Expression level and KS modifications, particularly by sulfation, fucosylation, and sialylation, significantly influence tissue function, and changes are frequently linked to the onset of different diseases such as macular corneal dystrophy, amyotrophic lateral sclerosis, Alzheimer’s disease, osteoarthritis, pancreatic cancer, and papillary thyroid carcinoma [26,27,28,29,30].

To gain a deeper understanding of the involvement of KS structure and function in health and disease, it is essential to employ highly specific tools to identify KS. Previously, we demonstrated that the mouse anti-KS monoclonal antibody (mAb) MZ15, directed against hepta- and octasaccharide KS oligosaccharides [31,32], is suited for in vitro molecular imaging by immunohistochemistry (IHC) and laser ablation inductively coupled plasma mass spectrometry (LA-ICP-MS) as a complementary method [33]. Moreover, other GAG-targeting antibodies have been used successfully for the detection of disease-related alterations in GAGs by immunofluorescence microscopy in the past [34,35,36,37].

Although mAbs are widely used for diagnostic applications, the use of antibody fragments and antibody-derived molecules such as single-chain variable fragments (ScFvs) can provide superior properties for target-specific molecular imaging [38]. A ScFv is a genetically engineered antibody fragment consisting of the variable-heavy (V_H_) and variable-light (V_L_) region domains of an antibody connected by a short and flexible peptide linker [39,40]. Despite their small size of approx. 26 kDa, ScFvs still retain the essential binding information of the original antibody [40,41]. Moreover, they can be cost-effectively produced in bacteria [39,41]. Although, several ScFvs targeting GAGs have been previously reported [42,43,44,45,46], to our knowledge, there is currently no KS-targeting ScFv reported.

Here, we describe the identification of the KS binding sequence of the mouse anti-KS mAb MZ15, the engineering, heterologous expression in *E. coli*, and purification of a functional anti-KS ScFv, derived from the parental mAb MZ15, and a workflow for the application of the ScFv for in vitro imaging of KS by immunofluorescence microscopy in rat eye cryosections as a model system.

## 2. Materials and Methods

### 2.1. Cultivation of MZ15 Hybridoma Cells and Production of MZ15 mAb

The cultivation of the murine hybridoma clone as well as the production and purification of the MZ15 mAb were performed as previously described [33].

### 2.2. Identification, Cloning, and Sequencing of the MZ15 Variable Regions

Amplification of the V_H_ and V_L_ genes of the MZ15 antibody was realized with a specific primer set described by Rohatgi et al. [47].

Cultivated MZ15 hybridoma (5 × 10^6^ cells) were harvested by centrifugation at 216× *g* for 10 min. Subsequently, total RNA was isolated using a Nucleospin RNA Mini Kit (Macherey Nagel, Düren, Germany) according to the manufacturer’s instructions. The isolated RNAs for the heavy and light chains, respectively, were then converted into cDNA using the M-MLV(H−) reverse transcriptase (Promega, Walldorf, Germany) and further amplified by PCR. A detailed protocol and a list of the primers used are given in the Supporting Information (see Tables S1–S6). Amplified PCR products were analyzed by agarose gel electrophoresis. Target bands at 479 bp for V_H_ and 397 bp for V_L_ were excised from the gel and purified using the Zymoclean Gel DNA Extraction Kit (Zymo Research, Freiburg, Germany). To assure a high accuracy in sequencing, the purified PCR products were initially cloned into the vector pBluescript II KS(+). To do so, the pBluescript II KS(+) was linearized with EcoRV-HF (New England Biolabs GmbH, Frankfurt am Main, Germany) for 30 min at 37 °C. Then, MZ15 V_H_ and V_L_ PCR products were inserted into the plasmid by T4 ligase. After the transformation of chemically competent *E. coli* DH5α, cells were plated on Luria–Bertani (LB) agar plates containing 100 µg/mL ampicillin. Transformants were screened for inserts and MZ15 variable genes identified by LightRun sequencing (Eurofins Genomics, Ebersberg, Germany). MZ15 V_H_ and V_L_ sequences were analyzed using IgBLAST [48]. A detailed cloning and ligation protocol is also described in the Supporting Information (see Table S7).

### 2.3. Construction of a ScFv Expression Vector and Protein Expression

The amino acid sequences for the V_H_ and V_L_ regions of the anti-keratan sulfate mAb MZ15, including the complementarity-determining regions (CDRs), were joined in silico by a 15-amino-acid linker region consisting of (Gly_4_Ser)_3_ in V_H_-(Gly_4_Ser)_3_-V_L_ orientation. Additionally, this sequence was flanked by NcoI (N-terminally) and XhoI (C-terminally) restriction sites, before it was inserted into a pET-22b(+) vector, containing a *pelB* leader sequence for the periplasmatic expression on the N-terminal site and further, a C-terminal 6xHis tag. This newly designed ScFv construct was first codon optimized for bacterial expression in *E. coli* and then de novo synthesized by BioCat GmbH (Heidelberg, Germany).

The de novo synthesized ScFv-pET-22b(+) vector was transformed into chemically competent *E. coli* BL21 (DE3) cells that were then plated and grown overnight on LB agar plates containing 100 µg/mL ampicillin at 37 °C. Single colonies were picked and inoculated in LB medium containing 100 µg/mL ampicillin and grown overnight at 37 °C and 150 rpm. This preculture was then re-inoculated in a larger volume of fresh LB medium containing 100 µg/mL ampicillin at an OD_600_ of 0.1 and further cultured at 37 °C and 150 rpm until an OD_600_ of 0.9–1 was reached. Afterwards, protein expression was induced by the addition of 1 mM IPTG and the culture was then further grown at 30 °C and 150 rpm for 4 h. Finally, cells were harvested by centrifugation at 6000× *g* and 4 °C for 30 min and cell pellets were stored at −20 °C until use.

### 2.4. Solubilization, Functional Refolding, and Purification of the ScFv

The harvested cell pellets of the ScFv expression in *E. coli* were resuspended in 20 mL 1× Tris-buffered saline (TBS), pH 7.4. Then, after adding a spatula tip of lysozyme and RNAse A, and 1× cOmplete protease inhibitor cocktail (Sigma-Aldrich Chemie GmbH, Taufkirchen, Germany), cells were disrupted by sonication with an overall “on” time of 5 min (30 s pulse, 1 min pause, 30% amplitude) on ice using a Branson SFX250 sonifier (Emerson Technologies GmbH & Co. OHG, Dietzenbach, Germany). Cell lysates were centrifuged at 30,000*× g* and 4 °C for 20 min. While supernatants were discarded, pellets were resuspended in 20 mL washing buffer containing 1× TBS, pH 7.4, 10 mM EDTA, 5 mM DTT, and 2% Triton X-100, and centrifuged afterwards at 15,000*× g* and 4 °C for 15 min, twice. Supernatants were discarded and pellets resuspended in 1× TBS, pH 7.4, 10 mM EDTA, and centrifuged again at 15,000*× g* and 4 °C for 15 min. The pellets were then once more resuspended in 1× TBS, pH 7.4, and 8 M urea and solubilized overnight at 4 °C under gentle rotation. After another centrifugation at 15,000*× g* and 4 °C for 15 min, the remaining supernatants were used for purification.

The purification of the denatured and solubilized 6xHis-tagged ScFvs was performed by nickel affinity chromatography. A total of 2 mL Cytiva Ni Sepharose High-Performance resin (Sigma-Aldrich Chemie GmbH, Taufkirchen, Germany) was filled into Econo-Pac Chromatography Columns (Bio-Rad, Feldkirchen, Germany) and equilibrated with 5 column volumes (CV) of binding buffer (1× TBS, pH 8.0, 6 M urea). After application of the solubilized sample, the column was washed with 10 CV washing buffer (50 mM Tris, pH 8.0, 500 mM NaCl, 6 M urea, 20 mM imidazole). Finally, bound ScFvs were eluted with 5 CV of elution buffer (50 mM Tris, pH 8.0, 500 mM NaCl, 6 M urea, 250 mM imidazole).

Subsequently, the purified but still denatured ScFvs were refolded by dilution of the Ni elution fraction in refolding buffer (1× TBS, pH 8.2, 1 mM EDTA, 2 mM reduced glutathione (GSH), 0.2 mM oxidized glutathione (GSSG), and 400 mM arginine). To do so, the Ni elution fraction was slowly dribbled by gravity at 4 °C into 20-fold of the elution fractions volume of cold refolding buffer using a syringe and a 0.40 × 0.2 mm cannula (B. Braun, Melsungen, Germany). The ScFv was then allowed to refold by further incubation of this solution at 4 °C for 40 h under gentle stirring. The solution was then centrifuged at 15,000*× g* and 4 °C for 15 min and filtered using a Steritop 0.22 µm bottle top vacuum filter (Merck KGaA, Darmstadt, Germany) to separate protein aggregates. The remaining filtered solution was concentrated, and buffer exchanged into 1× TBS, pH 7.4, using Amicon Ultra centrifugal filter units (Merck KGaA, Darmstadt, Germany) with a 10 kDa cut-off. The concentration of the purified MZ15-derived ScFv was determined spectrophotometrically by measuring the absorbance at 280 nm using a NanoDrop One/One^c^ UV-Vis spectrophotometer (Thermo Fisher Scientific, Schwerte, Germany). The theoretical molecular weight and extinction coefficient were calculated based on the amino acid sequence using the Expasy ProtParam tool (MW: 28,604 Da and ε: 42,650 M^−1^ cm^−1^). Sample purity and protein recovery was monitored by SDS-PAGE. Finally, aliquots of purified ScFvs were stored at 4 °C until use. The expression of the MZ15-derived ScFvs yielded in up to 5 mg protein/L culture.

### 2.5. KS Staining of Rat Eye Cryosections with the Single-Chain Variable Fragment

Frozen tissue sections of rat eyes were prepared as previously described [33]. Frozen rat eye cryosections were thawed and subjected to fixation and permeabilization using an ice-cold mixture of acetone/methanol (2:1) at −20 °C for 30 min. After allowing the solvent to evaporate, cryosections were encircled with a water-repellent ring and washed in 1× PBS, pH 7.4, three times for 5 min each. All subsequent steps were conducted in a humid chamber to prevent sample drying. To test for specific binding of the KS antigen by the MZ15-derived ScFv, some cryosections were exposed to 20–160 µg/mL *Niallia circulans* keratanase II in 1× TBS, pH 7.4, for 4 h at 37 °C. Details about the expression and purification of the *N. circulans* keratanase II are provided in the Supporting Information. After keratanase II digestion, cryosections were washed three times for 5 min each with 1× PBS, pH 7.4. To minimize nonspecific binding, cryosections were blocked with blocking solution containing 10% (*v*/*v*) normal goat serum (Merck, Darmstadt, Germany), 0.1% (*v*/*v*) Triton X-100 in 1× PBS, pH 7.4, for 2 h at room temperature (RT). During the blocking process, 10 µg/mL ScFvs in Antibody Diluent (Thermo Fisher Scientific, Schwerte, Germany) were pre-incubated with 10 µg/mL mouse anti-penta-His IgG mAb (QIAGEN, Hilden, Germany) in Antibody Diluent on ice for 2 h. After the cryosections were washed three times for 5 min each, they were covered with either the pre-incubated ScFv/mouse anti-penta-His IgG mAb-mixture or the MZ15 full-length mAb (MZ15 c_end_ = 1.7 µg/mL) in Antibody Diluent and incubated overnight at 4 °C. Hereafter, cryosections were washed another time with 1× PBS, pH 7.4, and incubated for 2 h at RT with Alexa Fluor 488-conjugated goat anti-mouse secondary antibody (ab150117, Abcam, Cambridge, UK), diluted to 1:200 in Antibody Diluent. Then, cryosections were washed again, before they were counterstained using DAPI (4′,6-diamidino-2-phenylindole, 1:10,000 in 1× PBS, pH 7.4). Finally, after washing the sections once more, they were mounted in Shandon Immu-Mount (Thermo Fisher Scientific, Schwerte, Germany). Immunofluorescence microscopy was performed using an Axio Observer Z1 (Carl Zeiss AG, Oberkochen, Germany) fluorescence microscope equipped with an AxioCam MRm camera. For image acquisition and processing, Axio Zen 3.7 software (Carl Zeiss AG, Oberkochen, Germany) was used.

## 3. Results

### 3.1. Identification of the V_H_ and V_L_ Genes from the Anti-Keratan Sulfate mAb MZ15

**Figure 1:**
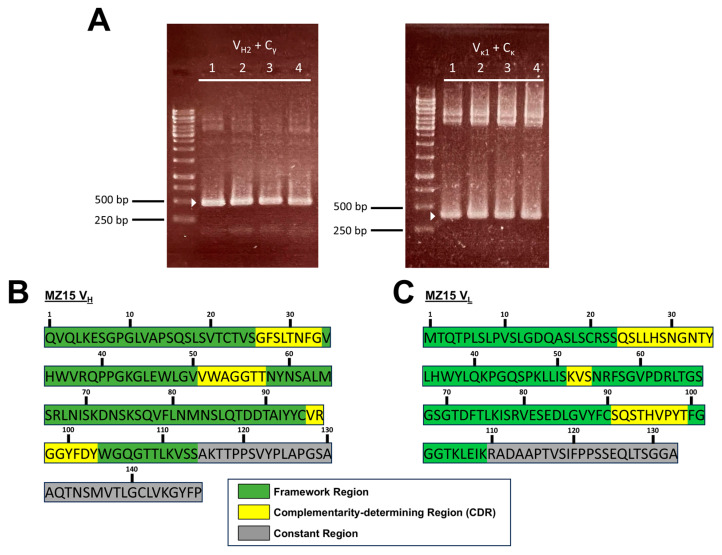
PCR products of amplification of mAb MZ15 V_H_ and V_L_ inside pBluescript II KS(+) and identified V_H_ and V_L_ amino acid sequences. (**A**) PCRs of MZ15 heavy-chain genes (**left**) and light chain genes (**right**). Target PCR product bands are visible at 479 bp for the heavy chain and 397 bp for the light chain, respectively (white triangles). After sequencing and in silico analysis using IgBLAST alignments, the variable region amino acid sequences of the mAb MZ15 (**B**) heavy chain and (**C**) light chain were revealed and functional antibody regions identified. Samples 1–4: selected clones. Original images of (**A**) can be found in Supplementary Materials.

Total RNA of the MZ15-producing hybridoma cells was isolated and converted to cDNA. To extract the genetic information from the MZ15 KS-binding paratope, we performed RT-PCR and PCR to convert the RNA into cDNA and amplify the respective V_H_ and V_L_ genes with a set of primers, previously described by Rohatgi et al. [47]. PCR products of expected sizes of 479 bp for the heavy chain and 397 bp for the light chain, respectively (Figure 1A), were subsequently cloned into the pBluescript II KS(+) vector. Four clones for each chain were picked, and DNA was extracted, sequenced, and further characterized in silico using the online tool IgBLAST. The resulting amino acid sequences encoded by the MZ15 V_H_ and V_L_ genes are shown in Figure 1B,C.

Analysis with IgBLAST allowed us to assign DNA and corresponding amino acid sequences to respective subdivisions of the variable regions. The framework regions (FRs), CDRs, and constant region were identified (see Figure 1B,C). Furthermore, variable region gene families could also be assigned. The MZ15 heavy chain was assigned to the IgHV2 gene family and the γ isotype, whereas the light chain belongs to the gene family IgκV1 and the kappa isotype.

### 3.2. Construction of a ScFv Expression Vector Based on the Anti-KS mAb MZ15

**Figure 2:**
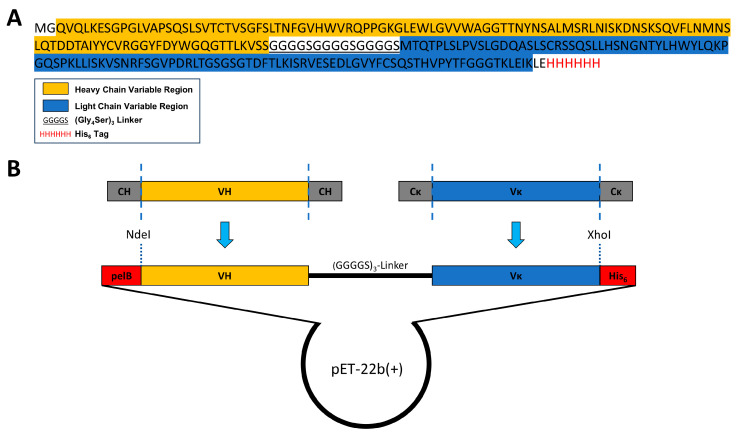
Expression construct. (**A**) Amino acid sequence and (**B**) design of the MZ15-derived ScFv expression vector for bacterial protein expression. The identified amino acid sequences of the mAb MZ15 V_H_ and V_L_ regions were joint together via a (Gly_4_Ser)_3_ linker. The artificial ScFv amino acid sequence was incorporated into the pET-22b(+) vector including a N-terminal *pelB* leader sequence for periplasmatic translocation and a C-terminal 6xHis tag essential for protein purification and utilization on tissue sections.

To assemble a single-chain variable fragment (ScFv), the V_H_ and V_L_ regions were joined with a flexible (Gly_4_Ser)_3_ linker. It has been shown that this configuration ensures a correct distance between both domains for proper folding of the ScFv [40,49]. The DNA construct was finally cloned into the *E. coli* expression vector pET-22b(+). A scheme of the ScFv expression vector and its amino acid sequence is shown in Figure 2.

Furthermore, this construct was generated with a N-terminal PelB leader sequence, a signal sequence inducing the translocation of the ScFv into the periplasm. The translocation of the ScFv into the bacterial periplasm is assumed to provide a beneficial oxidative environment for correct disulfide bond linking and thus folding of the ScFv [50,51,52]. Finally, after the DNA sequence of the final ScFv molecule had been codon optimized to ensure efficient protein expression in *E. coli*, the ScFv gene was de novo synthetized and incorporated into the pET-22b(+) vector.

### 3.3. ScFv Was Successfully Expressed and Purified from Inclusion Bodies

The pET-22b(+) vector, carrying the codon optimized sequence of the MZ15-derived ScFv, was transformed into *E. coli* BL21 (DE3). Under native conditions, we were not able to express the protein in a soluble fraction, but a massive amount was detected in inclusion bodies (IBs). We therefore decided to take this as the base material. The ScFv was isolated from the insoluble fraction following a protocol described earlier by Song et al. [53]. Accordingly, IBs were first solubilized under reducing conditions, with a strong chaotropic agent urea. Purification was performed with nickel affinity chromatography and the ScFv subsequently refolded by a 20-fold dilution in refolding buffer for 40 h, as described in [53,54]. Finally, the ScFv was concentrated and buffer exchanged to 1× TBS, pH 7.4, by using Amicon centrifugal filter units. During the concentration process, some protein aggregation was observed.

**Figure 3:**
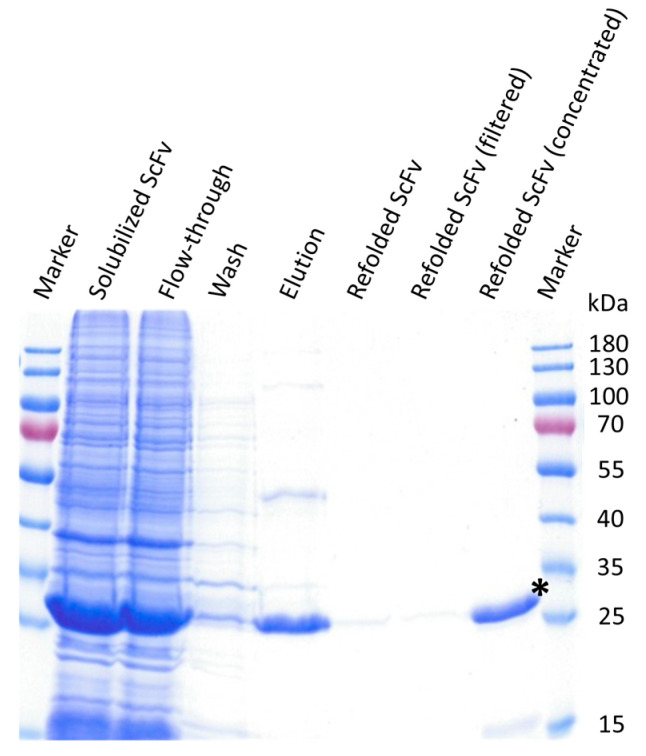
Anti-KS ScFv derived from mAb MZ15 expressed in *E. coli* BL21 (DE3). The insoluble fraction was solubilized under reducing conditions with 8 M urea (Solubilized ScFv). ScFv was then isolated by nickel affinity chromatography (Flow-through, Wash, Elution) and was subsequently refolded by a 20-fold dilution in refolding buffer (Refolded ScFv; Refolded ScFv (filtered)). Finally, the ScFv was concentrated and buffer exchanged to 1× TBS, pH 7.4, by using Amicon centrifugal filter units (Refolded ScFv (concentrated)). Samples taken at each step were separated by SDS-PAGE and Coomassie stained. The corresponding bands of the ScFv at 28.6 kDa (*) are clearly visible in the respective fractions. Original image can be found in Supplementary Materials.

Samples obtained during ScFv purification and refolding were visualized by SDS-PAGE and Coomassie Brilliant Blue staining (Figure 3). In the refolded and concentrated fraction, a clear band at about 28.6 kDa, corresponding with the theoretical molecular weight of monomeric ScFvs, is visible with a purity of about 89%, as assessed by scanning and densitometric analysis using ImageJ software v. 1.54 g. The purified soluble ScFv was finally stored at 4 °C until use.

### 3.4. ScFv Specifically Detects KS in Rat Eye Tissue Sections

**Figure 4:**
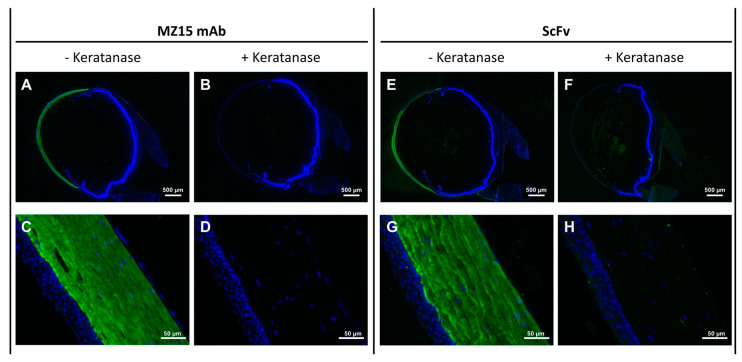
MZ15-derived ScFv and full-length antibody MZ15 exhibiting comparable performance in the detection of highly sulfated KS in rat eye frozen sections. Tissue sections underwent immunostaining, employing either the mAb MZ15 or a ScFv-anti-penta His mAb conjugate, followed by incubation with an Alexa Fluor 488-conjugated goat anti-mouse secondary antibody (green). Nuclear staining was achieved using DAPI (blue). Scale bars are provided within the images. Panels (**A**,**B**,**E**,**F**) give an overview, while panels (**C**,**D**,**G**,**H**) demonstrate the corneal stroma at higher magnification.

Localization of KS in the cornea of rat eye cryosections by immunofluorescence microscopy using the MZ15-derived ScFv was compared to that obtained when using mAb MZ15, previously shown to react specifically with KS in the rat cornea [33]. The ScFv was detected via the intrinsic hexa histidine tag with a mouse anti-penta His antibody that was reacted with the ScFv prior to application on the frozen rat eye tissue sections. After binding to the tissue section, the ScFv-mouse anti-penta His antibody conjugate could be detected using a fluorescently labeled anti-mouse antibody binding to the mouse anti-penta His antibody. In the frozen sections of the rat eye, a clear fluorescence signal was observed in the cornea, with a strong preference for the stroma (Figure 4E,G). An identical binding pattern was observed for the MZ15 full-length antibody (Figure 4A,C). In order to validate KS binding specificity, rat eye frozen sections were digested with *N. circulans* keratanase II prior to incubation with the ScFv-mouse anti-penta His antibody conjugate or with mAb MZ15, respectively. Digestion with keratanase II completely abolished the reactivity of the cornea with both the ScFv (Figure 4F,H) and the full-length mAb MZ15 (Figure 4B,D), demonstrating a comparable KS binding specificity. Control staining utilizing solely the anti-penta His antibody together with a fluorescently labeled secondary antibody gave no signal in the cornea (Figure S3).

## 4. Discussion

Studies on alterations in expression, composition, and localization of GAGs within ECMs are crucial to understanding the pathogenesis and course of diseases as well as regeneration processes. The identification of GAGs is often elegantly achieved by immunohistochemical methods using specific anti-GAG antibodies or antibody-derived molecules such as ScFv. ScFvs are advantageous as they can be produced cheaply in bacteria [39,41]. However, ScFvs generated so far were mostly directed against HS and CS [42,43,44,45,46]. To our knowledge a ScFv with binding specificity for KS has not yet been reported. Since KS is involved in many different types of disease, it was our aim to provide a ScFv targeting KS for imaging.

For the development of an anti-KS ScFv, the anti-KS mAb MZ15 was chosen as a base, since the binding of this mAb to KS had been thoroughly characterized [31,32]. Using various methods such as immunofluorescence and solid-phase radioimmunoassays, MZ15 was shown to preferentially react with sulfated hepta- and octasaccharide KS oligosaccharides and to have no cross-reactivity towards CS, DS, and hyaluronic acid [31,32]. The first step for the genetic engineering of a recombinant ScFv was the identification and amplification of the MZ15 full-length mAb V_H_ and V_κ_ genes. By performing RT-PCR, these genes were successfully amplified from MZ15 hybridoma cells using primers previously described by Rohatgi et al. [47]. The resulting DNA fragments were cloned into the pBluescript II KS(+) vector for subsequent sequential analysis. Using the online tool IgBLAST [48], FRs and CDRs were identified and heavy- and light-chain sequences assigned to variable regions IgHV2 and IgκV1, respectively. In this regard, a KS-binding paratope was identified. However, the functional relevance of key amino acids within the paratope for KS binding remains to be elucidated. This will be addressed in future work by introducing point mutations to the heavy- and light-chain variable regions.

Accordingly, the ScFv was designed using the following established and widely reported principles. The identified V_H_ and V_κ_ genes were merged with a 15-amino-acid-long flexible (Gly_4_Ser)_3_ linker in between [40,49,52,55,56]. The length and amino acid composition of the chosen linker were shown to be critical, as they influence the flexibility and relative formation and folding of both variable domains. The flexible (Gly_4_Ser)_3_ linker, with a length of 5.7 nm, was chosen to mediate a proper distance between V_H_ and V_κ_, facilitating functional monomeric folding and correct linkage of intra-chain disulfide bonds [40,49]. While cost-efficient bacterial protein expression is a significant advantage of ScFvs, the formation of insoluble and inactive protein aggregates, IBs, is a common feature. The formation of IBs during cytoplasmic expression is attributed to the reducing environment within the bacterial cytoplasm and the possibility for close interaction among only partially folded molecules during bacterial overexpression [52,57]. To overcome this, the ScFv gene was constructed with a N-terminal *pelB* leader sequence. PelB is a signal sequence that drives translocation into a bacterial periplasm. Due to its more oxidative environment, periplasmatic delivery might support correct disulfide bond assembly and ScFv folding [51,58]. Despite this, the expression of the recombinant ScFv-6xHis fusion protein in *E. coli* localized in the insoluble fraction. However, in several studies, ScFv from IBs could be functionally recovered by solubilization, denaturation, and subsequent refolding into soluble ScFv [52,53]. Likewise, the MZ15-derived ScFv could be recovered from the insoluble fraction. After solubilization and denaturation of the IBs, the ScFv was purified in an unfolded state by nickel affinity chromatography and subsequently refolded (Figure 3). Although protein aggregation occurred during concentration and buffer exchange, part of the ScFv remained in solution. Further optimization of the solubilization and refolding conditions, as well as the generation of MBP-ScFv fusion proteins, might significantly enhance the solubility, stability, and yield of the ScFv [52,59].

As a proof of concept, in vitro imaging of KS utilizing the MZ15-derived ScFv was performed on rat eye frozen sections as a model system. Frozen tissue sections offer the advantage of better preserving the natural structure of antigens, hence preserving their reactivity with antibodies more effectively [60]. Staining of rat eye frozen sections with the KS-targeting ScFv in combination with an intermediate anti-penta His mAb, and a fluorescence-labeled secondary antibody, revealed a clear fluorescence signal in the cornea of the rat eye. In that location, the fluorescence signal was primarily confined to the stromal region of the cornea. Among various tissues, the cornea is considered to contain the highest concentration of KS in the body [6,24,61,62]. Furthermore, the finding that KS was primarily detected in the cornea aligns with earlier observations made using the anti-KS mAb 5D4 [63,64] and our previous work using the mAb MZ15 along with mAb 5D4 [33]. As the identical pattern was observed with the mAb MZ15, the binding characteristics of the ScFv are in agreement with those of the mAb MZ15. In general, ScFvs are considered to maintain the binding specificity of their ancestral antibody [40,41]. The binding of the ScFv and mAb MZ15 to KS was validated by control experiments. In these experiments, the tissue sections were pre-digested with *N. circulans* keratanase II before exposure to either the ScFv or mAb MZ15, respectively. *N. circulans* keratanase II is an endo-*β*-*N*-acetylglucosaminidase that is able to degrade KS by cleavage of the *N*-acetylglucosamine linkage of KS, releasing mono- or disulfated Gal*β*1–4Glc*N*Ac disaccharides and several other oligosaccharides [65,66,67,68]. Enzymatic degradation of KS in the rat eye frozen sections completely abolished the reactivity of the ScFv and of mAb MZ15 (Figure 4B,D,F,H). This control verified that both the ScFv and mAb MZ15 specifically react with KS in the cornea. This finding is in full coincidence with the results of previous studies of using the mAbs MZ15 and 5D4 to detect KS in rat eye cryosections [22,31,32,33,69]. Both antibodies have been shown to be specific for sulfated poly-*N*-acetyllactosamine domains on KS oligosaccharides [22,31,32,33,69]. In the cornea, the synthesis and structural organization of the collagen matrix requires the sulfation of KS, making the cornea an ideal model system [70]. It is of interest to note that KS-binding antibodies have been shown to react with human-induced pluripotent stem (iPS) cells or embryonic stem (ES) cells [71], indicating that anti-KS mAb and ScFv may be used to examine the role of KS in iPS/ES cells.

## 5. Conclusions

In the present study, the binding paratope for KS determined by the mAb MZ15 was identified. On the basis of the paratope, a ScFv derived from the parental mAb MZ15 was designed and cloned. A workflow was established to produce the functional ScFv by expression in *E. coli*, isolation from the insoluble fraction by unfolding and nickel affinity chromatography, and subsequent refolding. As a proof of concept, the ScFv was successfully employed as an imaging tool for the immunofluorescence microscopy of KS in rat eye cryosections. With the aim of applying anti-KS ScFv for molecular in vitro and in vivo imaging of KS, the next steps will require detailed molecular characterization of the ScFv (protein structure, stability, affinity) in comparison with its parental mAb, direct labeling with suitable tracers, and characterization of the pharmacokinetics in vivo (clearance, distribution, serum half-life) of optimized ScFv constructs.

## Data Availability

The original contributions presented in this study are included in the article/supplementary material. Further inquiries can be directed to the corresponding authors.

## Supplementary Materials

The following supporting information can be downloaded at: https://www.mdpi.com/article/10.3390/biom15020178/s1, Figure S1: DNA sequences of the anti-KS mAb MZ15 variable regions; Figure S2: The 6xHis tag of the refolded MZ15-derived ScFv, fully accessible after solubilization of inclusion bodies, purification, and refolding of the ScFv; Figure S3: Staining of rat eye cryosections utilizing solely the anti-penta His mAb without ScFv, followed by incubation with an Alexa Fluor 488-conjugated goat anti-mouse secondary antibody; Figure S4: Soluble production of the *N. circulans* keratanase II in *E. coli* BL21 (DE3). Table S1: Isotype-specific primers for the reverse transcription reaction; Table S2: Composition of the reverse transcription reaction; Table S3: PCR primers for MZ15 heavy-chain amplification (from Rohatgi et al. [47]); Table S4: PCR primers for MZ15 light chain amplification (from Rohatgi et al. [47]); Table S5: Composition of the PCR for amplification of the isotype-specific genes of the MZ15 heavy- and light-chain variable region; Table S6: Program of the Touchdown PCR for amplification of the isotype-specific genes of the MZ15 heavy- and light-chain variable region; Table S7: Blunt-end ligation reaction composition for the insertion of the MZ15 variable region genes into pBluescript II KS(+). Original images of Figure 1A and Figure 3 can be found in Supplementary Materials.
