# Supplementary material for: Identification of an Immunoglobulin Paratope Binding to Keratan Sulfate and Expression of a Single-Chain Derivative for Imaging
Source: Biomolecules. 2025 Jan 25;15(2):178. doi: 10.3390/biom15020178 (PMC11852928; doi:10.3390/biom15020178)
Supplement: Supplementary file 1 [file biomolecules-15-00178-s001.zip › Supporting Information.pdf]

Supporting Information

Identification of an Immunoglobulin Paratope Binding to Keratan Sulfate and Expression of a Single-Chain Derivative for Imaging

Burak Boyraz [1,2], Rudolf Tauber [1,*] and Jens Dernedde [1,*]

[1] Institut für Laboratoriumsmedizin, Klinische Chemie und Pathobiochemie, Charité–Universitätsmedizin Berlin, Corporate Member of Freie Universität Berlin, Humboldt-Universität zu Berlin and Berlin Institute of Health, Augustenburger Platz 1, 13353 Berlin, Germany; boyrab92@zedat.fu-berlin.de
[2] Fachbereich Biologie, Chemie, Pharmazie, Freie Universität Berlin, Arnimallee 22, 14195 Berlin, Germany
* Correspondence: rudolf.tauber@charite.de (R.T.); jens.dernedde@charite.de (J.D.)

Identification of the heavy and light chain variable region of the mAb MZ15

Reverse transcription for conversion of total RNA into antibody isotype specific cDNA of the variable regions of MZ15 heavy and light chain

After isolation of total RNA from the MZ15 hybridoma cells, the isotype specific RNA encoding the variable region of the heavy and light chain of the mAb MZ15 were converted into cDNA by a reverse transcription. According to the Developmental Studies Hybridoma Bank (DSHB, Iowa, USA), where the hybridoma cells were initially obtained from, the heavy chain of the MZ15 mAb classifies to the mouse antibody subclass IgG1, while the light chain is determined as a kappa light chain (mAb MZ15 Datasheet, Developmental Studies Hybridoma Bank, Iowa, USA). Therefore, the following isotype specific primers (see Table S1) were used for the reverse transcription reaction (from Rohatgi *et al*. [1])

Table S1: Isotype specific primers for the reverse transcription reaction.

Antibody region specificity	Primer sequence (5'-3')
RTCγ (Heavy chain specific primer, IgG Isotype)	cagggatccaKagttc*
RTCκ (Light chain specific primer, kappa light chain)	tcaagaagcacacgac

* K = g/t

The reverse transcription reaction (25 µL) was composed as follows in Table S2:

Table S2: Composition of the reverse transcription reaction.

Ingredient	Quantity	c_{End} in 25 µL reaction volume
RNA	1 µg	1 µg
RT-primer (10 µM)	1.25 µL	0.5 µM
dNTPs (10 µM)	1.25 µL	0.5 µM
Reaction buffer (5x)	5 µL	1x
DTT (100 mM)	1 µL	5 mM
M-MLVRT(H-), Promega	1 µL	1 µL
Nuclease-free H_2O	13.5 µL	Ad 25 µL

The reverse transcription was performed at 42 °C for 1 h in two separate reactions for the heavy and light chain specific regions, respectively. The reverse transcriptase was then heat-inactivated by incubation at 99 °C for 5 min. After briefly cooling the reaction on ice, 2.5 µL RNase H Reaction Buffer (10x, Thermo Fisher Scientific, Schwerte, Germany) and 1 µL RNase H (5 U/µL, Thermo Fisher Scientific, Schwerte, Germany) were added to the solutions and further incubated for 1 h at 37 °C to degrade remaining RNA.

Amplification of the heavy and light chain variable region genes of the mAb MZ15

The unpurified cDNA was further used as template in the following touchdown PCR for specific amplification of the variable regions of the MZ15 heavy and light chain. For the PCR, primers also described by Rohatgi *et al.* (2008) were used, which are listed below in the following tables Table S3 and Table S4 [1]. Since our final aim is the elucidation of the sequences of the heavy and light chain of the mAb MZ15, only the external sets of primers from Rohatgi *et al.* (2008) were used [1]. Because the gene families of the MZ15 heavy and light chain regions were not known, heavy and light chain specific primers were pooled for a first amplification approach to V_H1 – V_H16 and $V_\kappa1$ – $V_\kappa19$, respectively, where each primer was added to the mix at a concentration of 2 µM. On the other hand, the reverse primers for the constant region of the IgG heavy chain C_γ were not pooled but instead have been used in two separate reactions. The exact composition of the Touchdown PCR is shown in Table S5. The

Touchdown PCR has been performed according to the program listed in Table S6 using an Applied Biosystems 2720 Thermal Cycler (Thermo Fisher Scientific, Schwerte, Germany).

Table S3: PCR primers for MZ15 heavy chain amplification (from Rohatgi *et al.* [1]).

IgG Heavy Chain Primers

Gene family	Primer (5'-3')	Degeneration
V_H1	agRtYcagctgcaRcagtct	R: a/g; Y: c/t
	aggtccaactgcagcagcc	
V_H2	tctgcctggtgacWttccca	W: a/t
V_H3	gtgcagcttcaggagtcag	
V_H4	gaggtgaagcttctcgagtc	
V_H5	gaagtgaagctggtggagtc	
V_H6	atgKacttgggactgaRctgt	K: g/t; R: a/g
V_H7	cagtgtgaggtgaagctggt	
V_H8	ccaggttactctgaaagagtc	
V_H9	tgtggaccttgctattcctga	
V_H10	tgttggggctgaagtgggttt	
V_H11	atggagtgggaactgagctta	
V_H12	agcttcaggagtcaggacc	
V_H13	caggtgcagcttgtagagac	
V_H14	atgcagctgggtcatcttctt	
V_H15	gactggatttggatcacKctc	K: g/t
V_H16	tggagtttggacttagttggg	
C_γ	agggaaataRcccttgaccag	R: a/g
	agggaagtagcctttgacaag	

Table S4: PCR primers for MZ15 light chain amplification (from Rohatgi *et al.* [1]).

Igκ Light Chain Primers

Gene family	Primer (5'-3')	Degeneration
$V_{\kappa}1$	tgatgacccaRactccact	R: a/g
$V_{\kappa}2$	gcttgtgctctggatccc	
$V_{\kappa}3$	ctgctgctctgggttcc	
$V_{\kappa}4$	cagcttcctgctaatcagtg	
$V_{\kappa}5$	ctcagatccttggacttHtg	H: a/c/t
$V_{\kappa}6$	tggagtcacagacYcagg	Y: c/t
$V_{\kappa}7$	tggagtttcagacccagg	
$V_{\kappa}8$	ctgctMtgggtatctggt	M: a/c
$V_{\kappa}9$	cWtcttgttgctctggtttc	W: a/t
$V_{\kappa}10$	gatgtcctctgctcagttc	
$V_{\kappa}11$	cctgctgagttccttggg	
$V_{\kappa}12$	ctgctgctgtggcttaca	
$V_{\kappa}13$	ccttctcaacttctgctct	
$V_{\kappa}14$	agggcccYtgctcagttt	Y: c/t
$V_{\kappa}15$	atgagggtccttgctgag	
$V_{\kappa}16$	gaggttccaggttcaggt	
$V_{\kappa}17$	ccatgaccatgYtctcact	Y: c/t
$V_{\kappa}18$	atggaaactccagcttcattt	
$V_{\kappa}19$	atgagaccgtctattcagtt	
C_{κ}	gcacctccagatgttaactg	

Table S5: Composition of the PCR for amplification of the isotype specific genes of the MZ15 heavy and light chain variable region

Ingredient	Inserted volume in 50 µL reaction
Phusion HF reaction buffer (5x)	10 µL
Pooled primer mix for VH or Vκ (2 µM primer master mix)	10 µL
Pooled primer mix for Cγ or Cκ (2 µM stock)	2.5 µL
dNTPs (10 mM stock)	1 µL
Unpurified cDNA	5 µL
Phusion HotStart II polymerase (2 U/µL)	1 µL
Nuclease-free H_2O	20.5 µL

Table S6: Program of the Touchdown PCR for amplification of the isotype specific genes of the MZ15 heavy and light chain variable region

Reaction step	Temperature [°C]	Time [min]
Initial denaturation	98	2
**Touchdown cycles (5 cycles, 2 °C decreasing of annealing temperature, each)**		
Denaturation	98	1
Annealing	62 - 52	1
Elongation	72	1
**30x steady cycles**		
Denaturation	98	1
Annealing	62 - 52	1
Elongation	72	1
Final elongation	72	10
Hold	4	∞

PCR products were separated by a 1.5% agarose gel. Bands at 479 bp for the heavy chain and 397 bp for the light chain (Figure S1), respectively, were cut out. DNA was purified using the Zymoclean Gel DNA Recovery Kit (Zymo Research Europe GmbH, Freiburg, Germany). Purified gene products were sequenced by LightRun sequencing (Eurofins Genomics, Ebersberg, Germany). Finally, heavy and light chain sequences were analyzed using IgBLAST and assigned to the antibody gene families and isotypes. However, sequencing short gene products yield inaccurate sequences. Therefore, after identifying the gene families, the procedure was repeated, by using the specific primers of the identified gene family instead of the pooled primer mix to generate the PCR product, as previously described in this section. In this specific instance, the primers for the V_H2 gene family, along with both $C_γ$ primers in separated reactions, respectively, were used for the heavy chain, and the primers for the $V_κ1$ gene family were used in conjunction with $C_κ$ for the light chain.

A

MZ15 VH

```
TCTGCCTGGTGACATTCCCAAGCTGTGTCCTGTCCCAGGTGCAGCTGAAGGAGTCAGGACCTGGCCTGGTGGCGCCCTCACAGAGCCTGTCCGTCACTTG  < 100
         10        20        30        40        50        60        70        80        90

CACTGTCTCTGGATTTTCATTAACCAACTTCGGTGTCCACTGGGTTCGCCAGCCTCCAGGAAAGGGTCTGGAGTGGCTGGGAGTAGTATGGGCTGGTGGA  < 200
        110       120       130       140       150       160       170       180       190

ACCACAAATTATAATTCGGCTCTCATGTCCAGACTGAACATCAGTAAAGACAACTCCAAGAGTCAAGTTTTCTTAAACATGAACAGTCTACAAACTGATG  < 300
        210       220       230       240       250       260       270       280       290

ACACAGCCATATACTACTGTGTCAGGGGGGGCTACTTTGACTACTGGGGCCAAGGCACCACTCTCAAAGTCTCCTCAGCCAAAACGACACCCCCATCTGT  < 400
        310       320       330       340       350       360       370       380       390

CTATCCACTGGCCCCTGGATCTGCTGCCCAAACTAACTCCATGGTGACCCTGGGATGCCTGGTCAAGGGTTATTTCCCT  < 479
        410       420       430       440       450       460       470
```

B

MZ15 Vκ

```
TGATGACCCAGACTCCACTCTCCCTGCCTGTCAGTCTTGGAGATCAAGCCTCCCTCTCTTGCAGATCTAGTCAGAGCCTTTTACACAGTAATGGAAACAC  < 100
         10        20        30        40        50        60        70        80        90

CTATTTACATTGGTACCTGCAGAAGCCAGGCCAGTCTCCAAAGCTCCTGATCTCCAAAGTTTCCAACCGATTTTCTGGGGTCCCAGACAGGCTCACTGGC  < 200
        110       120       130       140       150       160       170       180       190

AGTGGATCAGGGACAGATTTCACACTCAAGATCAGCAGAGTGGAGTCTGAGGATCTGGGAGTCTATTTCTGCTCTCAAAGTACACATGTTCCGTACACGT  < 300
        210       220       230       240       250       260       270       280       290

TCGGAGGGGGGACCAAGCTGGAAATAAAACGGGCTGATGCTGCACCAACTGTATCCATCTTCCCACCATCCAGTGAGCAGTTAACATCTGGAGGTGC  < 397
        310       320       330       340       350       360       370       380       390
```

Figure S1: DNA sequences of the anti-KS mAb MZ15 variable regions. (**A**) Base sequence of the heavy chain variable region with a length of 479 bp. (**B**) Base sequence of the light chain variable region with a length of 397 bp.

Cloning of MZ15 variable region PCR gene products into pBluescript II KS(+)

In the repeated experiment, the obtained gene product was cloned into the plasmid pBluescript II KS(+) as follows to enhance sequencing accuracy. First, 1.7 µg pBluescript II KS(+) was linearized by incubation with 1 µL EcoRV-HF (10 U/µL, New England Biolabs GmbH, Frankfurt am Main, Germany) for 30 min at 37 °C. Linearized pBluescript II KS(+) was separated by a 1% agarose gel electrophoresis, cut out and purified by the Zymoclean Gel DNA Recovery Kit (Zymo Research Europe GmbH, Freiburg, Germany). With the MZ15 heavy and light chain specific PCR products and the linearized pBluescript II KS(+) a blunt-end ligation was performed in a 3:1 insert/vector molar ratio as described in Table S7.

Table S7: Blunt-end ligation reaction composition for the insertion of the MZ15 variable region genes into pBluescript II KS(+)

**Linearized vector DNA**	100 ng
**Insert DNA (3:1)**	43.57 ng
**10x T4 DNA ligase buffer (Thermo Fisher Scientific, Schwerte, Germany)**	2 µL
**50% PEG 4000**	2 µL
**T4 ligase (5 U/µL, Thermo Fisher Scientific, Schwerte, Germany)**	1 µL
**Nuclease-free water**	Ad 20 µL

The ligation mixtures were incubated overnight at 16 °C. Afterwards, 5 µL ligation sample was transformed into chemically competent *E. coli* DH5α cells and plated on Luria-Bertani (LB) agar plates containing 100 µg/mL ampicillin. Plated bacteria were incubated overnight at 37 °C.

Validation of 6xHis tag accessibility of the MZ15-derived ScFv after refolding

Accessibility of the 6xHis tag of the purified and refolded MZ15-derived ScFv was tested by nickel affinity chromatography. 800 µL Cytiva Ni Sepharose High Performance resin (Sigma-Aldrich Chemie GmbH, Taufkirchen, Germany) were filled into Econo-Pac Chromatography Columns (Bio-Rad, Feldkirchen, Germany). First, the resin was equilibrated with 5 CV nickel binding buffer (1x TBS, pH 7.4). After loading of the refolded ScFv onto the column, washing was performed using 10 CV nickel binding buffer (1x TBS, pH 7.4). Bound protein was eluted by application of 5 CV nickel elution buffer (1x TBS, pH 7.4, 250 mM imidazole). All fractions of purification were concentrated 50-fold using Amicon Ultra centrifugal filter units (Merck KGaA, Darmstadt, Germany) with a 3 kDa cut-off. The proportion of bound ScFv with accessible 6xHis tag was monitored by SDS-PAGE.

The 6xHis tag of the refolded MZ15-derived ScFv is fully accessible and functional

The MZ15-derived ScFv was designed with a C-terminal 6xHis tag to allow simple purification of the molecule, and since this tag is an ideal target for the detection of the bound ScFv in imaging studies e.g. in tissue sections. Therefore, the accessibility and functionality of the 6xHis tag of the refolded ScFv was validated by nickel affinity chromatography (Figure S2).

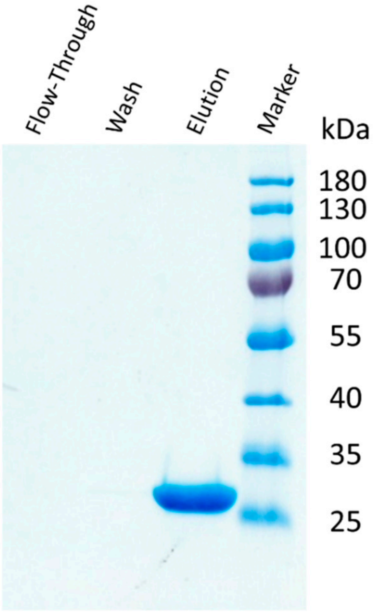

Figure S2: The 6xHis tag of the refolded MZ15-derived ScFv is fully accessible after solubilization of inclusion bodies, purification, and refolding of the ScFv. SDS-PAGE of the fractions obtained after nickel affinity chromatography of the refolded ScFv shows a clear band at about 28.6 kDa exclusively inside the eluate fraction.

When the refolded ScFv was applied to the nickel affinity column a strong polypeptide band at about 28.6 kDa was visible only in the elution fraction indicating that the 6xHis tag of the refolded ScFv is fully accessible and functional after the purification procedure under denaturing conditions and subsequent refolding of the molecule.

Testing for unspecific staining of the anti-penta-His mAb in rat eye cryosections

The mouse anti-penta-His IgG mAb (QIAGEN, Hilden, Germany) was utilized as intermediate antibody during the staining of rat eye cryosections with the MZ15-derived ScFv. As the Fc region of a mouse antibody is no longer included in the developed ScFv, preventing the use of fluorescence labelled anti-mouse secondary antibodies, the anti-penta-His antibody was utilized to bridge between the 6xHis tagged ScFv and the fluorescence labelled secondary antibody. Control stainings on rat eye

cryosections using solely the mouse anti-penta-His IgG together with an Alexa Fluor 488-conjugated goat anti-mouse secondary antibody had no effect on the corneal stroma (Figure S3).

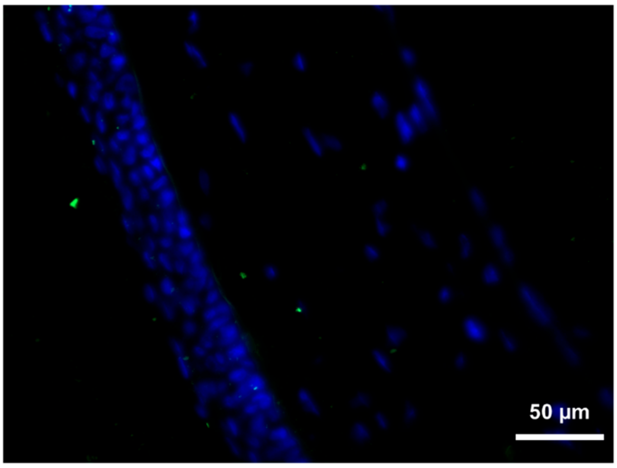

Figure S3: Staining of rat eye cryosections utilizing solely the anti-penta His mAb without ScFv, followed by incubation with an Alexa Fluor 488-conjugated goat anti-mouse secondary antibody. No fluorescence signal was detected in the corneal stroma. Nuclear staining was achieved using DAPI (blue). Scale bar is given within the image.

Cloning, Expression and purification of a *Niallia circulans* keratanase II

The keratanase II from *N. circulans* (formerly *Bacillus circulans*) is an endo-*β*-*N*-acetylglucosaminidase, able to cleave the *N*-acetylglucosamine bond within KS, resulting in the release of mono- or disulfated Gal*β*1−4Glc*N*Ac disaccharides and various longer oligosaccharides [2-4]. Availability of *N. circulans* keratanase II was important to remove the epitope from respective tissue slides to prove KS binding specificity of the ScFv. As the enzyme is not commercially available the gene was cloned and the enzyme recombinantly expressed in *E. coli*.

The DNA sequence of the *N. circulans* keratanase II gene (GenBank accession no.: AY188989.1) was used as template and modified according to the work of Wang *et al.* [5] to generate a truncated keratanase II variant. The modified enzyme is characterized by the absence of the D domain and by a curtailed C-terminus after amino acid residue 1502. It was demonstrated that these modifications notably enhance both enzyme activity and stability. The DNA base sequence was codon optimized for bacterial expression in *E. coli* and de novo synthesized by General Biosystems, Inc. (Durham, North Carolina, USA). The keratanase II sequence was flanked by NdeI (N-terminally) and XhoI (C-terminally) restriction sites, together with a C-terminal 6xHis tag and inserted into a pET-22b(+) vector without a *pel*B leader sequence for cytoplasmatic expression.

For the expression of the keratanase II, first, the pET-22b(+) vector harboring the keratanase II sequence was transformed into chemical competent *E. coli* BL21 (DE3) cells. Transformed cells were plated and grown on LB agar plates containing 100 µg/mL ampicillin overnight at 37 °C. Precultures were made by picking single colonies, inoculating LB medium containing 100 µg/mL ampicillin and growing these cultures overnight at 37 °C and 150 rpm. Then, main cultures for bacterial enzyme expression were made by re-inoculating the precultures at a starting OD_{600} of 0.1 into a larger volume of fresh LB medium containing 100 µg/mL ampicillin and further incubating these at 37 °C and 150 rpm until an OD_{600} of 0.6 was reached. Enzyme expression was induced by the addition of 0.5 mM IPTG and cultures then further grown at 30 °C and 150 rpm for 4 h. Cells were harvested by centrifugation at 6,000 x g and 4 °C for 30 min and cell pellets were stored at -20 °C until use.

In the following, cell pellets were resuspended in 15 mL 1x TBS, pH 7.4. After a spatula tip of lysozyme and RNAse A, and 1x cOmplete protease inhibitor cocktail (Sigma-Aldrich Chemie GmbH, Taufkirchen, Germany) has been added to the cell suspensions, cells were disrupted by sonication with an overall “on”-time of 5 min (30 s pulse, 1 min pause, 30 % amplitude) on ice using a Branson SFX250 sonifier (Emerson Technologies GmbH &. Co. OHG, Dietzenbach, Germany). To separate cell debris from

soluble proteins, lysated cell suspensions were centrifuged at 30,000 x g and 4 °C for 20 min. Pellets were discarded and the remaining supernatants were further used for purification of the enzyme.
The keratanase II cloned into the pET-22b(+) leads to the production of an enzyme having a C-terminal 6xHis tag enabling a one-step purification by nickel affinity chromatography. Therefore, 2 mL Cytiva Ni Sepharose High Performance resin (Sigma-Aldrich Chemie GmbH, Taufkirchen, Germany) were filled into Econo-Pac Chromatography Columns (Bio-Rad, Feldkirchen, Germany). After the nickel resin has been equilibrated with 5 CV nickel binding buffer (1x TBS, pH 7.4), the amylose elution fraction was directly applied to the column. Then, washing was performed with 10 CV nickel washing buffer (1x TBS, pH 7.4, 20 mM imidazole). For the elution of the keratanase II from the nickel resin, 5 CV nickel elution buffer (1x TBS, pH 7.4, 250 mM imidazole) was applied to the column. The resulting elution fraction was concentrated, and buffer exchanged into 1x TBS, pH 7.4 using Amicon Ultra centrifugal filter units (Merck KGaA, Darmstadt, Germany) with a 50 kDa cut-off.
Enzyme concentrations were determined spectrophotometrically by measuring the absorbance at 280 nm utilizing a NanoDrop One/One^c UV-Vis spectrophotometer (Thermo Fisher Scientific, Schwerte, Germany). The calculation of the theoretical molecular weights and extinction coefficients of the keratanase II was performed based on its amino acid sequence using the Expasy online tool ProtParam. Accordingly, the following theoretical values were used: MW: 157481 Da and ε: 279270 M^{-1} cm^{-1}. Purity of the sample and protein recovery was monitored by SDS-PAGE (Figure S4). Aliquots of 6xHis tagged keratanase II were snap-frozen using liquid nitrogen and stored at -80 °C until use.

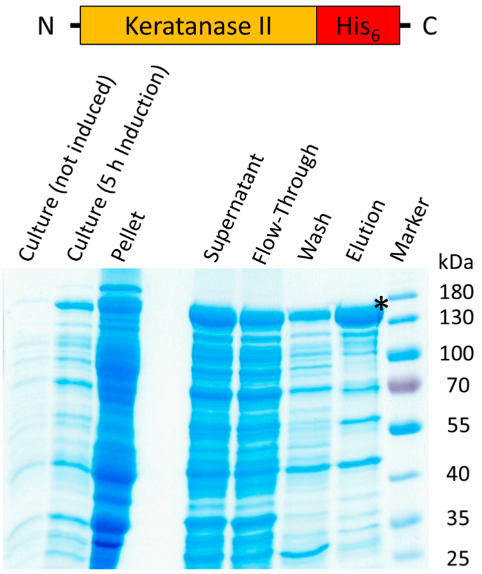

Figure S4: Soluble production of the *N. circulans* keratanase II in *E. coli* BL21 (DE3). A 6xHis tagged keratanase II was produced utilizing the pET-22b(+) vector and purified by nickel affinity chromatography. After SDS-PAGE and Coomassie staining a corresponding protein band is clearly visible at about 157 kDa (*).

The keratanase II could be successfully purified with a very high yield of 30 mg enzyme/L cell culture and a purity of 46%, as assessed by densitometric analysis of scanned gel bands. The enzyme was successfully utilized for removal of KS epitopes in our previous work [6].

References

1. Rohatgi, S., P. Ganju, and D. Sehgal, *Systematic design and testing of nested (RT-)PCR primers for specific amplification of mouse rearranged/expressed immunoglobulin variable region genes from small number of B cells.* Journal of Immunological Methods, **2008**. 339(2): p. 205-219.
2. Brown, G.M., T.N. Huckerby, H.G. Morris, B.L. Abram, and I.A. Nieduszynski, *Oligosaccharides derived from bovine articular cartilage keratan sulfates after keratanase II digestion: implications for keratan sulfate structural fingerprinting.* Biochemistry, **1994**. 33(16): p. 4836-46.

3. Brown, G.M., T.N. Huckerby, and I.A. Nieduszynski, *Oligosaccharides derived by keratanase II digestion of bovine articular cartilage keratan sulphates.* Eur. J. Biochem., **1994**. 224(2): p. 281-308.

4. Yamagishi, K., K. Suzuki, K. Imai, H. Mochizuki, K. Morikawa, M. Kyogashima, K. Kimata, and H. Watanabe, *Purification, Characterization, and Molecular Cloning of a Novel Keratan Sulfate Hydrolase, Endo-β-N-acetylglucosaminidase, from Bacillus circulans *.* J. Biol. Chem., **2003**. 278(28): p. 25766-25772.

5. Wang, H., W. He, P. Jiang, Y. Yu, L. Lin, X. Sun, M. Koffas, F. Zhang, and R.J. Linhardt, *Construction and functional characterization of truncated versions of recombinant keratanase II from Bacillus circulans.* Glycoconjugate Journal, **2017**. 34(5): p. 643-649.

6. Boyraz, B., J. Saatz, I.-M. Pompös, M. Gad, J. Dernedde, A.-K.B. Maier, O. Moscovitz, P.H. Seeberger, H. Traub, and R. Tauber, *Imaging Keratan Sulfate in Ocular Tissue Sections by Immunofluorescence Microscopy and LA-ICP-MS.* ACS Applied Bio Materials, **2022**. 5(2): p. 853-861.
