# Supplementary figures and images for: Identification of an Immunoglobulin Paratope Binding to Keratan Sulfate and Expression of a Single-Chain Derivative for Imaging
Source: Biomolecules. 2025 Jan 25;15(2):178. doi: 10.3390/biom15020178 (PMC11852928; doi:10.3390/biom15020178)

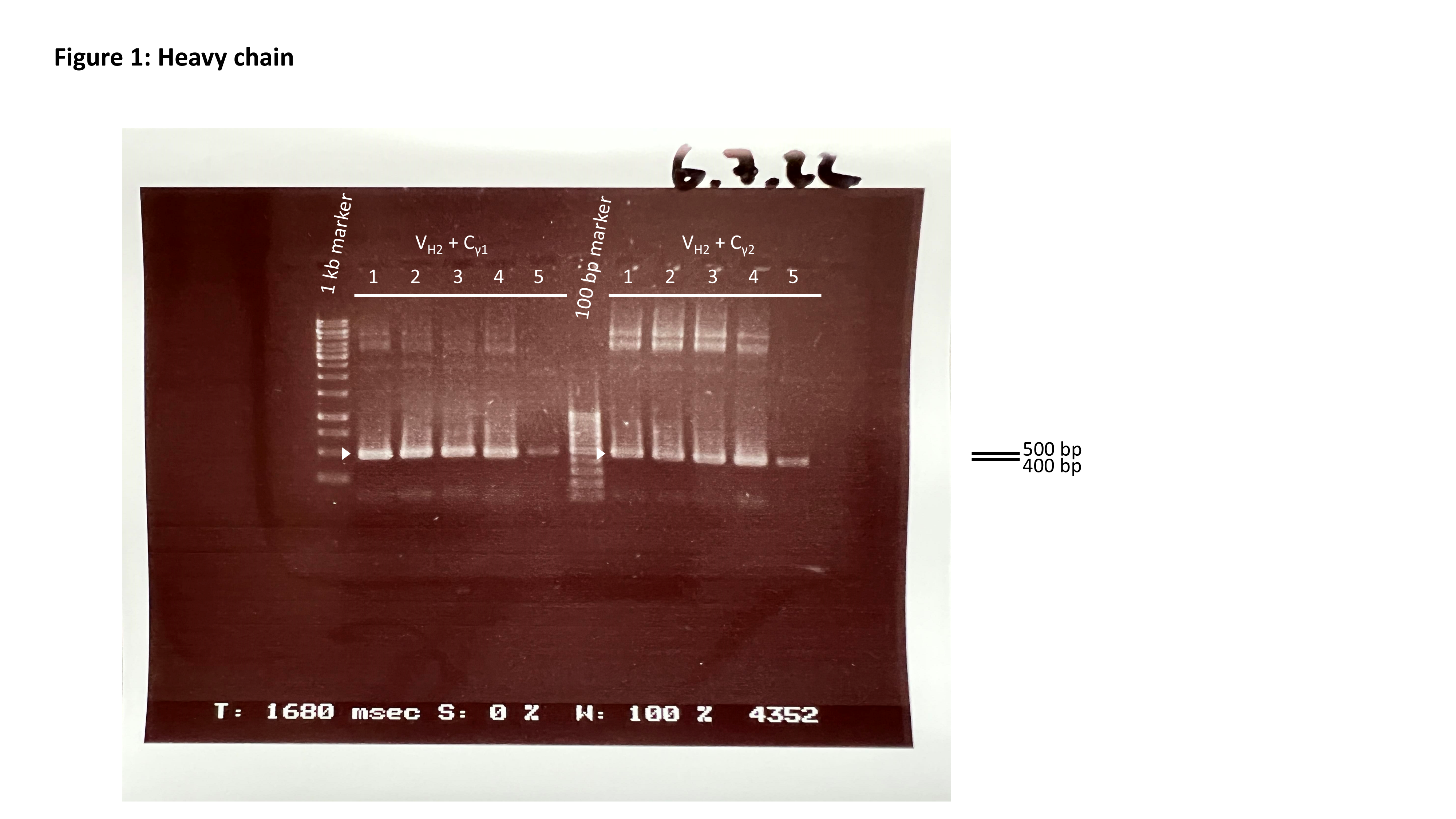

Supplement: Supplementary file 1 [file biomolecules-15-00178-s001.zip › biomolecules-3285436-original-images/Raw Gels/Figure 1 Heavy Chain.tif]

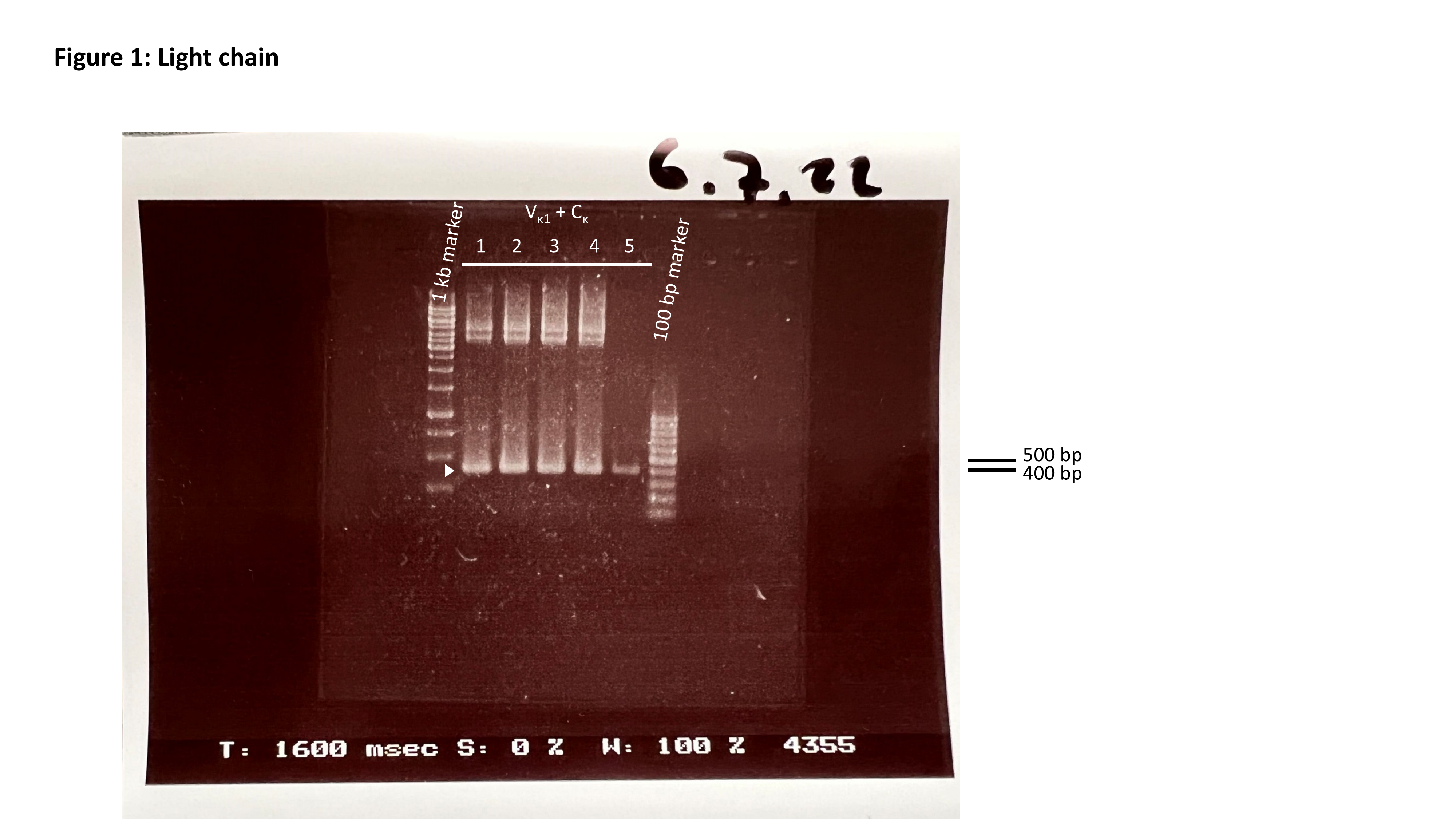

Supplement: Supplementary file 1 [file biomolecules-15-00178-s001.zip › biomolecules-3285436-original-images/Raw Gels/Figure 1 Light Chain.tif]

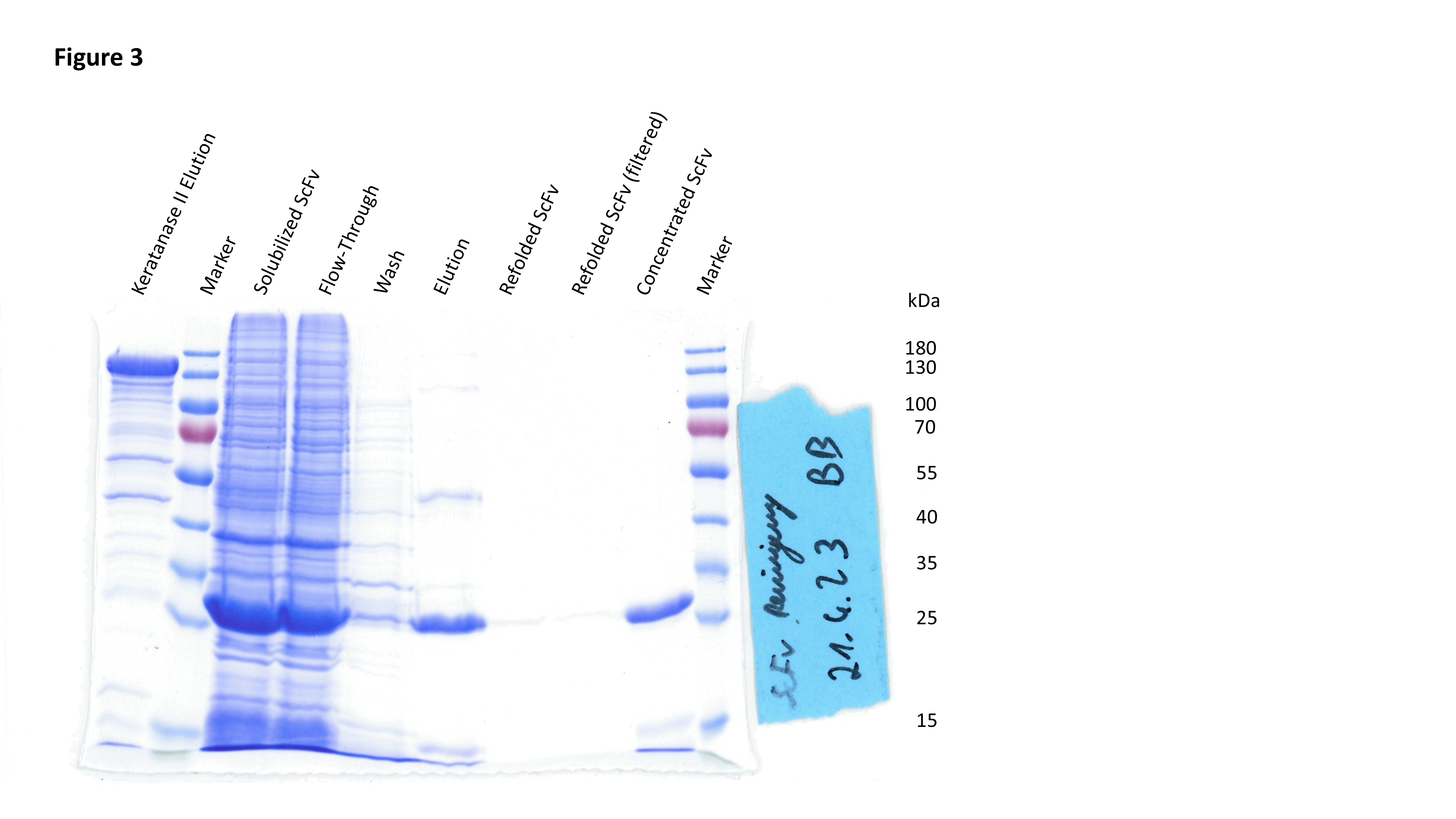

Supplement: Supplementary file 1 [file biomolecules-15-00178-s001.zip › biomolecules-3285436-original-images/Raw Gels/Figure 3.tif]

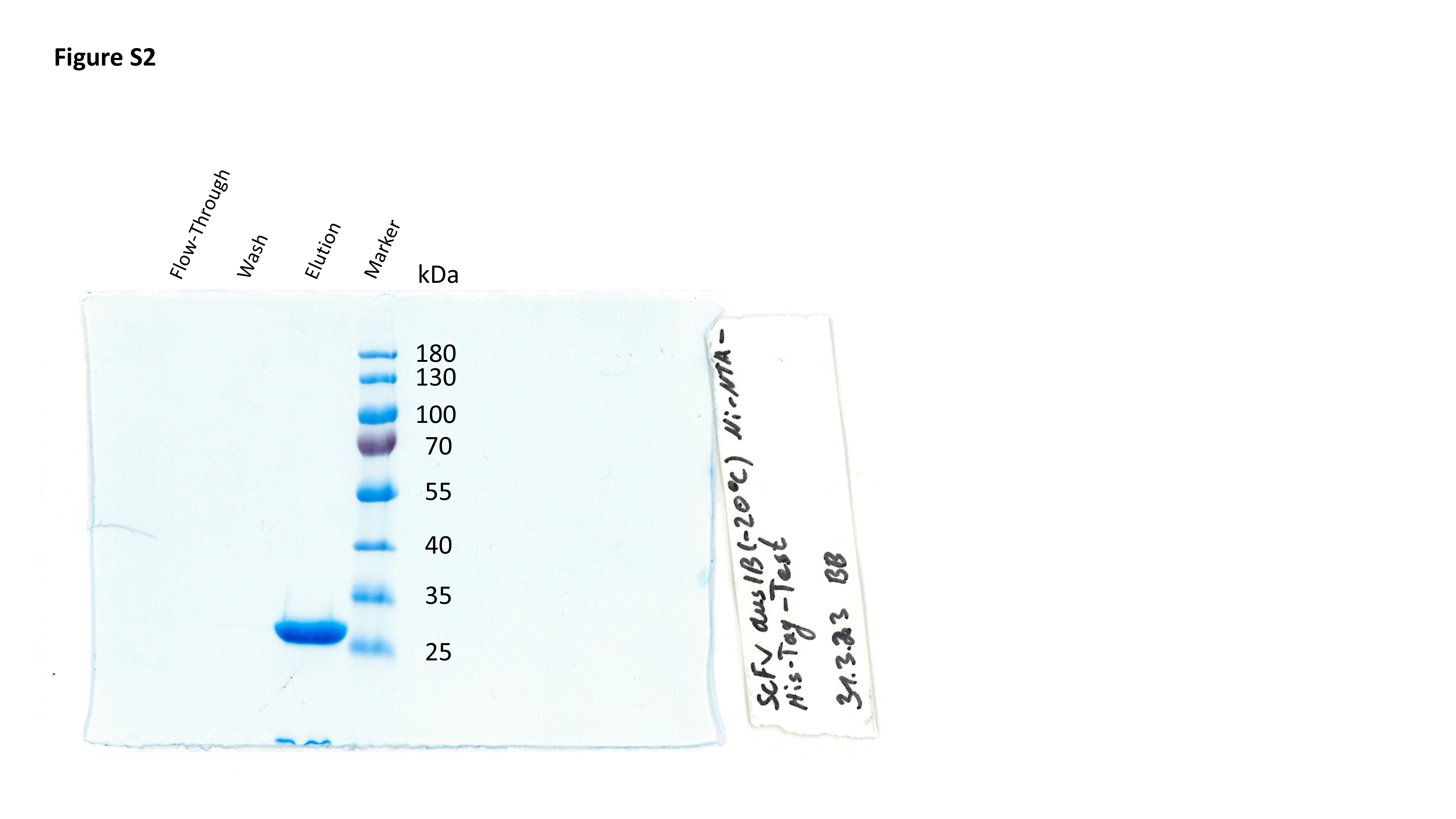

Supplement: Supplementary file 1 [file biomolecules-15-00178-s001.zip › biomolecules-3285436-original-images/Raw Gels/Figure S2.tif]

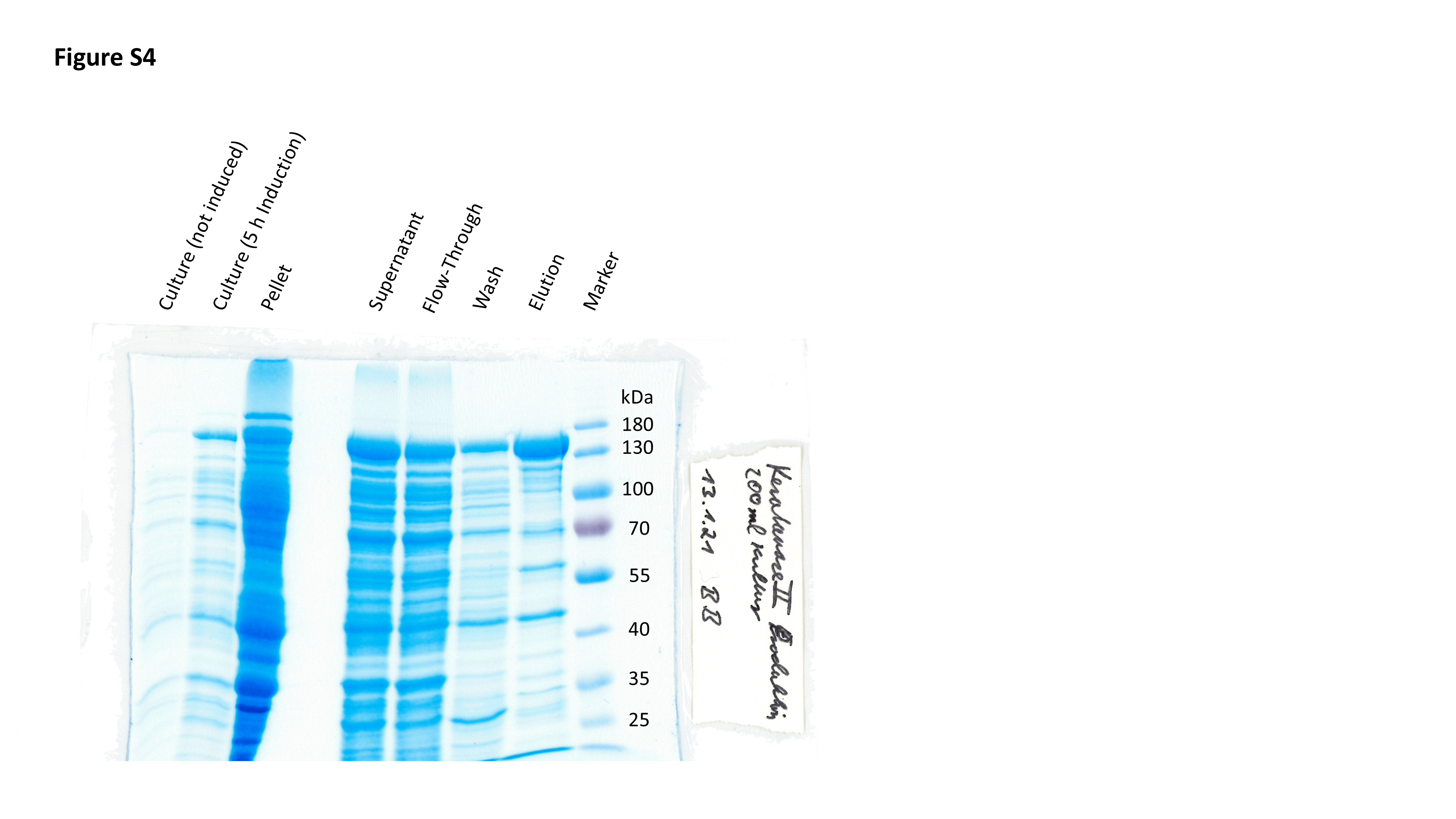

Supplement: Supplementary file 1 [file biomolecules-15-00178-s001.zip › biomolecules-3285436-original-images/Raw Gels/Figure S4.tif]
